# The role of professional logics in quality register use: a realist evaluation

**DOI:** 10.1186/s12913-020-4944-x

**Published:** 2020-02-11

**Authors:** Ann-Charlott Norman, Mattias Elg, Annika Nordin, Boel Andersson Gäre, Beatrix Algurén

**Affiliations:** 1 Jönköping Academy for Improvement of Health and Welfare, Jönköping University, Box 1026, SE-551 11 Jönköping, Sweden; 2 Department of Management and Engineering, HELIX Competence Centre, Linköping University, Linköping, Sweden; 3 Futurum, Academy for Health and Care Region Jönköping County, Ryhov County Hospital, Jönköping, Sweden; 4 Department of Food and Nutrition, and Sport Science, Faculty of Education, University of Gothenburg, Gothenburg, Sweden

**Keywords:** Quality registers, Programme, Clinical practice, Professional logics, Realist evaluation

## Abstract

**Background:**

Clinical practice improvements based on quality-register data are influenced by multiple factors. Although there is agreement that information from quality registers is valuable for quality improvement, practical ways of organising register use have been notoriously difficult to realise. The present study sought to investigate the mechanisms that lead various clinicians to use quality registers for improvement.

**Methods:**

This research involves studying individuals’ decisions in response to a Swedish programme focusing on increasing the use of quality registers. Through a case study, we focused on heart failure care and its corresponding register: the Swedish Heart Failure Register. The empirical data consisted of a purposive sample collected longitudinally by qualitative methods between 2013 and 2015. In total, 18 semi-structured interviews were carried out. We used realist evaluation to identify contexts, mechanisms, and outcomes.

**Results:**

We identified four contexts – *registration, use of output data, governance,* and *improvement projects* – that provide conditions for the initiation of specific mechanisms. Given a professional theoretical perspective, we further showed that mechanisms are based on the logics of either *organisational improvement* or *clinical practice*. The two logics offer insights into the ways in which clinicians choose to embrace or reject certain registers’ initiatives.

**Conclusions:**

We identified a strong path dependence, as registers have historically been tightly linked to the medical profession’s competence. Few new initiatives in the studied programme reach the clinical context. We explain this through the lack of an organisational improvement logic and its corresponding mechanisms in the context of the medical profession. Implementation programmes must understand the logic of clinical practice; that is, be integrated with the ways in which work is carried out in everyday practice. Programmes need to be better at helping core health professionals to reach the highest standards of patient care.

## Background

Policy officials, decision makers, and clinicians agree that there is an urgent need to make better use of register data in clinical practice. Although information from registers is generally considered valuable, practical ways of organising register use have been notoriously difficult to realise [1]. Although there is evidence of some clinical organisations using registers, competence in improvement based on registers is generally low [2]. Doubts have been raised about whether registers are used for evaluating services at clinical practice level [3]. The central problem is therefore not registers as such, but how to make them useful in clinical practice.

For quality registers to function properly, people engaged in their use need to cooperate with the various interests of local clinical and politico-administrative leadership, as well as professional colleagues, to achieve successful use [4–6]. We argue that there is significant tension between these different interests, which makes the various groups’ engagement in quality registers in clinical practice more or less successful. The various tensions are historically grounded. Over a long period, quality registers have developed from being a clinical support tool developed foremost for research purposes by the medical profession, to becoming a specific domain of interest with its own resources, success measures, and structural arrangements [7]. Therefore, quality registers are situated in a context of two types of logics: people engaged in use of quality registers and professionals working in clinical practice. These two groups differ in their forms of professionalism based on varying assumptions, values, beliefs, and rules that shape their work [8].

The purpose of the present study is to identify mechanisms that lead various groups within clinical healthcare organisations to use quality registers for improvement. Mechanisms refer to the underlying drivers behind the reasoning and decisions of a particular group [9]. By undertaking our analysis in the context of realist evaluation and theory of professional work, we contribute knowledge about the implementation of policy initiatives in large-scale healthcare systems.

This research involves studying individuals’ decisions in response to a Swedish programme focusing on increasing the use of National Quality Registers (NQRs). We focused on heart failure care and its corresponding register: the Swedish Heart Failure Register (SwedeHF).

## Methods

### The National Quality Register programme – the setting of the study

**Fig. 1:**
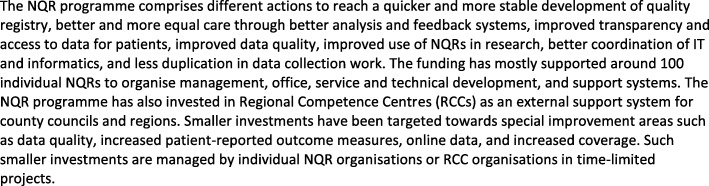
Details of the Swedish National Quality Register (NQR) programme

The Swedish National Quality Register (NQR) programme (see Fig. 1 for details) is part of a national policy agreement. A central aim of this policy agreement has been to increase the use of NQR data in efforts to improve healthcare.

We chose to study SwedeHF because it has a relatively short history and is therefore less established than other registers. SwedeHF began registering patient data in 2003. It collects disease- and treatment-specific information on individuals with heart failure. The effect of funding from the NQR programme was expected to be more obvious in SwedeHF than in a well-established, long-developed register. In 2016, approximately 50 hospitals and 66 primary healthcare centres participated in SwedeHF [10].

### Design and sample

The empirical data consisted of a purposive sample collected longitudinally by qualitative methods between 2013 and 2015, namely 18 semi-structured interviews within SwedeHF. Furthermore, the use of SwedeHF was studied in one specific context: a university hospital in one of the three biggest regions in Sweden.

The individuals interviewed were chosen using theoretical and concept sampling [11]. Representatives from NQR contexts as well as the healthcare contexts were interviewed. Additional file 1 describes the data collection process and gives a detailed overview of the purposeful sampling. Additional file 2 is a translation of the two interview guides that were developed and used for this particular study.

### Research process

Analysis started with a thematic categorisation of the empirical data [12]. We roughly categorised contextual factors that facilitated or governed the use of SwedeHF in clinical practice. We then used realist evaluation as an analytical tool to identify underlying mechanisms and their relationships to contexts and outcomes [9]. Each identified mechanism was described with an overall meaning and a significant citation from the empirical material, see Table 1. Furthermore, we used a theory of professionalism [13, 14] to categorise the identified mechanisms according to whether they belong within either an *organisational improvement logic* or a *clinical practice logic*. An important function of this categorisation is to elicit how and to what extent mechanisms are present in various contexts of register activities. The identified mechanisms are further classified according to six aspects of professionalism: people tend to *focus* (1) their work and base their achievements on various forms of *competenci*es (2); people at work are also monitored through different types of *control systems (3) and* base their actions on *specific motives (4);* people link their work to different *development rationales (5);* and the *type of work* (6) is also a means of understanding an individual’s capacity for certain actions. Given this theory of professionalism, the analysis contributes with new perspectives on the main question of the realist evaluation agenda: what works for whom and in which context? Additional file 3 provides a detailed description of how theory and methodology inform each part of the research process.

**Table 1:** Empirically identified mechanisms that prompt the use of SwedeHF information in clinical work

	Mechanisms grounded in…					
	… organisational improvement			… clinical practice		
	*Mechanism*	*Meaning*	*Illustrative quote*	*Mechanism*	*Meaning*	*Illustrative quote*
Focus	Performance improvement	SwedeHF^a^ supports feedback on performance in various forms. Feedback, then, aims to trigger improvement.	“We learn from our colleagues // but [SwedeHF helps] also to lift our eyes and look up….”	Individual patient’s health improvement	SwedeHF data help healthcare practitioners improve the health conditions of individual patients.	“It may be a bit of fun when filling in medicine doses. The first visit had the lowest doses, and when we send them out they are up to target doses.”
Competence	Improvement competence	Competence in improvement relates to SwedeHF measurements and how they can be embedded in improvement activities.	“The development of working methods and approaches can be neglected if you only focus on the academic. You need skills in change management and quality improvement work.”	Professional competence	SwedeHF has historically been tightly linked to the medical profession’s competence and the use of data is also due to the professional’s identification with the register.	“The indicators we use nowadays cannot be affected by nurses. It is primarily a doctor’s action that can improve the results. But healthcare is a collective effort, so some decisions from us can be partially affected by a nurse.”
Forms of control	Part of the job	Activated as a result of SwedeHF data being part of formal job descriptions.	“I sometimes do it online but that’s because… it’s pretty fast for me, because I already know what to look for. But we encountered some resistance from colleagues who thought, ‘No, not another thing to do’.”	Professional authority	SwedeHF activities through the legitimisation of leaders from the professional domain (e.g., senior MDs).	“Physicians are very critical of whether it will gain power or impact. // It’s their commitment that is the most important thing.”
Motives	P4P^a^ – Incentives	Use of the SwedeHF is motivated by financial initiatives.	“If there is no financial incentive, then I do not think it can be implemented in the way we wish. As long as it’s voluntary, people can say I’m not doing it because of my workload.”	Social control	SwedeHF, and its operation in clinical practice, signal to the organisation’s members that its use is important.	“At a clinic, there is only one who [person] is interested; the others don’t give a damn. It is never possible to do good registry work. // The boss must signal that we prioritise registry work, then people start trying to group themselves. This atmosphere at the clinic, as far as register work is concerned, is positive.”
Development rationale	Adaptation to society’s development	SwedeHF initiated through its role in creating transparent, resource-efficient healthcare.	“To avoid things like delayed treatment and examination and so forth. So, I think it’s the beginning to make it look good. As long as you use quality indicators to control healthcare, it starts with giving feedback on some form of improvement.”	Career enhancement	Professionals’ engagement in SwedeHF activity provides the potential for career enhancement.	“She [resident physician] has already shown her work and she has probably also come up with suggestions for how to improve this. And I have presented it at another meeting // And we have also sent out … an email reminder to all doctors that this and that will be in the care summary.”
Type of work	Organisational improvement	Activities whereby individuals carry out quality improvement-related work that is to be embedded in clinical work.	“It often awakens a lot of thoughts, so there’s a lot we can do to improve it [SwedeHF] and to make the information a tool throughout the patient’s care process.”	Occupational improvement	Focus on everyday clinical work and the embeddedness of SwedeHF.	“I say we have a form that we always review. Is it okay if we look at it? // And then it will not be as dramatic when I ask: how much do you drink*?”*

^a^*SwedeHF* Swedish Heart Failure Register, P4P: Pay for Performance

## Results

First, we provide an overview of the various mechanisms identified, and then present four empirical contexts that provide conditions for the initiation of certain mechanisms.

### Mechanisms that prompt the use of register data

Identification and categorisation of the mechanisms of register data use show that groups of people that mainly work in organisational improvement differ in action and rationale from those whose work is based on clinical practice. Thus, the professional logics offer insights into the ways in which people choose to embrace or reject certain register initiatives.

### Contexts of register data use

**Fig. 2:**
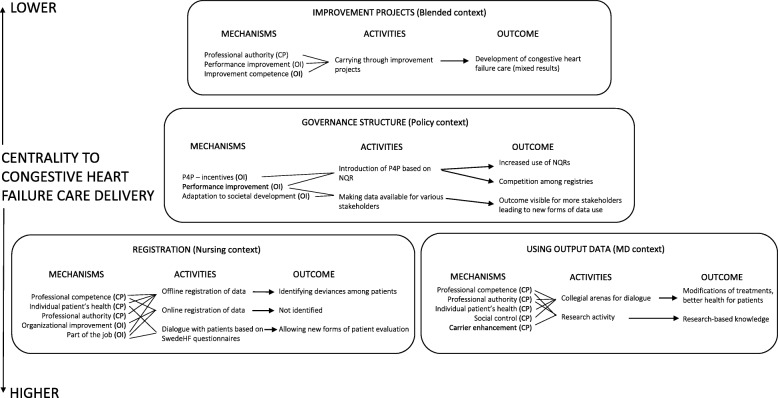
Four organisational contexts where the identified mechanisms activate specific outcomes in clinical practice (OI = organisational improvement; CP = clinical practice)

The mechanisms presented in Table 1 provide the foundation for understanding drivers behind the reasoning of different groups in their use of register data. However, these mechanisms only work if the circumstances are right. That is, the mechanisms are activated under certain contextual conditions. This section presents four empirically identified organisational contexts in the operation of various mechanisms and outcomes of SwedeHF use in heart failure care. The four organisational contexts are: *registration*, *using output data*, *governance structure*, and *improvement projects*; see Fig. 2.

In each context, we have identified specific activities and outcomes that are related to the NQR programme. More specifically, as the mechanisms are linked to either *organisational improvement* or *clinical practice*, we were able to understand what type of register activity worked for whom in what context.

### Registration – a nursing context

The NQR programme has the objective of improving forms of registration of data. Three activities are identified from this context: new ways of carrying out offline registration, new ways of carrying out online registration, and new forms of dialogue with the patient based on questionnaires.

### Offline registration

Offline registration is initiated through at least four mechanisms: professional competence, concerns for the individual’s health, being part of the job, and professional authority.

The mechanism of professional competence is of particular interest since registration is considered to require specific competencies, not just the ability to enter numbers into a database. Doctors do not use the questionnaires as they register patient information in medical records, whereas nurses and assistant nurses subsequently document entries in the register as part of their jobs. A clinic’s doctor who is responsible for SwedeHF^1^ states that it is a problem that doctors do not add data to the register:

“Ideally, doctors should register the data…because then the doctor gets this direct ‘aha experience’…. [however] We have too small an organisation, so doctors do not get the opportunity.”

Thus, the job requires professional competence for nurses or medical doctors. The assistant nurses are perceived to be unable to interpret data on registration, so they refer to nurses instead:

“It’s not me who works with it because I’m just a ‘register registrar’, but it will be the other nurses who have these vulnerable patients and they meet with the [doctors]*.*” (Registration assistant nurse)

Professional authority is also important in the registration context. There is a broad substantiated consensus among the respondents that SwedeHF is a medical register, which is why only the doctors can make a difference in practice by taking action based on the results. Central to the professional authority mechanism is the fact that nurses have access to the work of medical doctors (MDs). Whenever nurses identify deviances when working with the register, there is a need for authority to speak with the MDs. A registration nurse, when entering the register, reacted to worsening results as follows:

“… If I go to [the doctor responsible for SwedeHF], she may in turn request a printout… What did it look like last year? Yes, last year we were good at it and now suddenly we’re not. What happened? And then she has to take it to her doctors’ group.”

A clinic’s doctor responsible for SwedeHF described the same situation based on her medical perspective:

“Yes, the nurse… can react to something and then they come and tell me that this seems to say so and so. But no more than that.”

### Online registration

During the NQR programme, online registration was introduced as a new way of capturing data about the patient. This registration occurs during the patient meeting. Regardless of how promising this initiative seems to be, it was generally considered to involve a lot of difficulties. Decisions to complete the online registration were based on mechanisms linked to professional competence, organisational improvement, and being part of the job. A nurse, who for some time had the task of retroactively registering data, expressed that the online register was an opportunity, but her colleagues did not see the same possibility. The reasons she uses the register is explained by her professional competence, combined with the fact that she believes that SwedeHF registration is part of her job.

“I sometimes do it online, but that’s because it’s pretty fast for me, because I already know what to look for. But we encountered some resistance where some of my colleagues thought ‘No, not another thing to do.’”

### Dialogue with patients based on questionnaires

One positive consequence of new registration activities is that they create new ways of talking with patients, which enables the identification of health problems which may have previously been difficult to detect, such as questions about alcohol use. The mechanisms that make this possible are the healthcare personnel’s concerns for the individual patient and the fact that they can also refer to these questions as part of their clinical practice, as well as part of their job with organisational improvement. A nurse elaborates on the reasons for this:

“I say we have a form that we always review. Is it okay if we look at it? // And then it will not be as dramatic when I ask: how much do you drink*?”*

From a different perspective, a medical information director emphasised the role of NQRs as a means for asking questions in dialogue with the patient:

“And when I read through the questions, for example for bipolar disorder or for mental illness, I think if we do not ask those questions, I think we abdicate our medical mission; it’s about how to handle firearms, there are risks to others, are there risk situations? So, for me I think using NQRs might well be a means of support in the conversation with the patients.”

### Mechanisms in the registration context – a summary

The mechanisms that trigger actions in the registration context are based on the logic of both clinical practice and organisational improvement. What seems to be present here is that the professional registration work is carried out by nurses. MDs who, ideally, should carry out the registration, do not have the time, and assistant nurses do not have the professional competence to perform an informed registration. Consequently, the learning that could have emerged from registering data is not utilised sufficiently. A new form of online registration is met with scepticism, and the most important mechanisms that prompt registration are nurses with a broad level of competence and involvement in the SwedeHF register.

### Using output data – an MD context

At the core of organisational improvement is the use of data. Therefore, the issue is not only about choosing, defining, and designing data in registers, but also about finding out how the results should be used in practice. Therefore, the context of using output data is central. When empirically studying this organisational context, we identify two distinct types of activities: participating in collegial arenas for dialogue about patients and conducting research activities.

### Participating in collegial arenas for dialogue about patients

The organisational context of output data is the medical doctor’s domain, as SwedeHF only comprises medical indicators that can be handled by MDs. One central activity is participating in collegial arenas where individual patients and patient groups are discussed. The results from these activities are modifications of treatments with the aim of creating better health for patients. The mechanisms that trigger this activity are professional competence, professional authority, concerns about an individual patient’s health, and social control. The belief that SwedeHF data mainly require a medical doctor’s competence and authority is clearly argued by a professor and chief physician, who states that the results need to be mainly improved by doctors, but that teamwork with nurses is important:

“The indicators we use nowadays cannot be affected by nurses. Without that, it is primarily a doctor’s action that can improve the results. But healthcare is a collective effort. So, some decisions from us can be partially affected by a nurse.”

The empirical data in the present study indicate that the use of output data has not changed much during the NQR programme.

### Engaging in research activity

In addition to doctors meeting on a regular basis to discuss results from the SwedeHF, they also engage in research activity. A clinical manager elaborates on the importance of a research culture in order to use NQRs in the organisation’s development:

“If you have a culture… of engaging in research, clinical research, then you also have a culture of being fully up to date with all research carried out in the outside world and acquiring new data and new findings and being prepared to modify the treatment.”

One mechanism of particular importance is career enhancement. A clinic’s doctor who is responsible for SwedeHF describes how a medical resident worked with data from SwedeHF and how it is spread at the clinic:

“She [resident physician] has already shown her work and she has probably also come up with suggestions for how to improve this. And I have presented it at another meeting. // And we have also sent out then… an email reminder to all doctors that this and this will be in the epicrisis.”

The mechanisms in the output data context arise from clinical practice. Initiatives to drive organisational improvement in this context are not present.

### Governance structure – a policy context

NQRs are currently shifting from being an area of interest for professionals to becoming a tool for transparent public reporting, resource allocation, and administrative control. Therefore, the use of quality registers is also seen in the organisational context of governance. By governance, we mean the ways in which NQRs are handled and used by policy officials and administrators with a leading position in the healthcare system. In the case study material, respondents discuss the benefits and pitfalls of pay-for-performance (P4P) and transparent public reporting based on NQR data. In this section, we present the mechanisms that prompt these activities and specific outcomes.

### Introduction of P4P

The introduction of P4P has led to an increased focus on NQRs. It has also led to increasing competition between registers. The mechanisms that activate NQR use in this context are P4P incentives and performance improvement.

A clinical manager takes a positive view of goal-related compensation for NQR measurements, although he can also see certain risks:

“Yes, I think it’s good that you request it so clearly and that you connect resources. Even if it hurts a little bit if you fail. // It becomes very clear that it is important. Even from the governing level so to speak, then… but there is a risk of treatment. Instead of treating patients, you treat your data, so to speak.”

### Making data available to various stakeholders

Many NQRs have increasingly been made available to the public. This has led to results being visible to more stakeholders and new forms of data use, especially with a focus on patient processes and results. The mechanisms behind this are a general trend for adaptation to societal development and performance improvement. A healthcare director elaborates on making data more transparent for patients:

“It is like a trend in society as a whole, and in healthcare in general, to be better at following up and we are beginning to get more on PROM^2^ and PREM^3^ measurements*.*”

The respondents active in the governance context emphasise the use of registers for various stakeholders. However, the tendency within medical doctors’ groups is to resist this development. This is elaborated upon in the next section.

The context of governance is largely driven by mechanisms arising from the organisational improvement logic. We cannot identify any mechanisms based on the clinical practice logic; on the contrary, initiatives in this context are resisted in many ways. A medical director of information describes a professional commitment to improving conditions for the patient, but when the development is driven by policymakers, doctors become sceptical:

“Most doctors, yes, almost all I would say, are interested in developing and improving conditions for the patient. That is not a problem. And you want to find new forms of care, you want to improve quality, but there will be some kind of conflict in using this to save money or increase productivity. Productivity is a sad concept in healthcare. ‘How will these numbers be used?’ [doctors ask].”

We have identified that medical leadership is not communicating with the governance structure. As we saw in the context of output data, NQR use has only undergone minor developments during the programme period. Why have NQRs not led to more of an impact? One central aspect is the lack of medical leadership in the governance structure. Medical leadership involves the mechanism of professional authority, which activates NQR use through the legitimisation of leaders from the professional domain (such as senior MDs). However, medical leadership for NQRs is also related to the job carried out by formal leaders in the medical domain. Below we present empirical evidence of the troublesome role of medical leadership in the administration of SwedeHF.

A nurse who believes that it is a leader’s job to act on results said:

“Yes, I assume that… the head of the clinic [laughs] is looking at this. I have not asked him [the head], I only… the material is there. Seems crazy not to use it then. Then he could see, for example, that the prescribing of a particular drug is very low, contrary to the guidelines. And then it’s his responsibility as a medical leader to go to his doctors’ group and say… get to it! [laughs]”

One administrator, who has a background as a physician, says it is difficult to reach the clinical practice from her level:

“It’s not always easy to know which level to start with… you end up somewhere in the middle, and then you may find a supporter, but it does not mean that there is backing for it throughout the organisation.”

### Improvement projects – a blended context

A fourth organisational context is improvement projects. This context is blended, in the sense that activities and outcomes are generated by mechanisms stemming from both organisational improvement and clinical practice logics. The mechanism bundle of professional authority, performance improvement, and improvement competence initiates activities for improvement.

We see mixed outcomes in the improvement projects that were carried out in relation to the NQR programme. The presence or absence of professional authority explains this mechanism. Two nurses, who took part in an improvement project that was considered a failure, illustrate this problem:

“Our colleagues do not feel at all motivated to fill in these lists, because they see no gains whatsoever, or that they get anything back. We have always struggled to get a doctor, a medical officer to join us, but we have not succeeded, and then it is very difficult to move on.”

“It feels like our doctors are not interested at all in documenting.”

We also identified *improvement competence* – that is, know-how about conducting improvement work – as an important mechanism for activating successful improvement projects. In another improvement project, technical engineers (builders of the register) met with professional users as well as national managers of the SwedeHF register. The improvement project became a learning arena in which the participants gained new insights and understanding from each other, which in turn developed how they, with each perspective, proceeded with the progress and implementation of SwedeHF. Consequently, the improvement project became a catalyst for integrating SwedeHF into clinical practice. The interviewed national managers of SwedeHF referred to this quality improvement arena when they eventually became convinced that improvement competence is important for increased use in practice. They were initially sceptical about the improvement that projects could contribute based on their earlier understanding of research benefits.

At the heart failure clinic, there was an apparent division in views between the quality improvement leaders and the clinicians. Although they both had involvement in the SwedeHF, the two groups did not talk about the register and its benefits in the same way. The clinical manager was aware of and concerned by this division, especially as he predicted a risk that the professionals could lose interest in using the SwedeHF. He did not want them to stop using the register as it was seen as important for clinical improvement. Simultaneously, the clinical manager described the need for improvement skills in relation to science and professional knowledge:

“Organisational improvement can be so much more than science. And maybe more in those [scientific] areas of an organisation, we need some kind of improvement skills, yes. Otherwise, we have a tradition in academic healthcare provision of just pursuing that scientific development*.*”

Activities in the improvement context are initiated through a combination of three central mechanisms: professional authority, performance improvement, and improvement competence. These mechanisms originate from different logics, and here we see a potential for integrative efforts.

### Summary of results

The empirical analysis has identified four different organisational contexts that have been influenced by the NQR programme, to various extents:

- Registration – this is mainly a nursing context in which old and new forms of registration activities are carried out. The activities aim to support medical doctors’ activities in the use of output data from the SwedeHF. The mechanisms emanate from the clinical practice logic as well as the organisational improvement logic.
- Use of output data – a context in which medical doctors use information from the SwedeHF for decision-making. The use of information takes on different forms such as direct contact with nurses, dialogue forums with colleagues, and research activities. The lack of formal leadership of SwedeHF register use is notable. The mechanisms prompting NQR activity mainly emanate from the clinical practice logic.
- Governance – a context of policymakers and administrators, some of whom have medical backgrounds. During the programme, motives for deepened use of NQRs are practised through P4P initiatives. This context also involves activities for making data from NQRs more widely available. The mechanisms that drive activity are based on organisational improvement logics. Doctors in clinical contexts tend to oppose initiatives in this direction.
- Improvement projects – a blended context that is influenced by mechanisms from both organisational improvement logics and clinical practice. We see mixed outcomes in the improvement projects that were carried out in relation to the NQR programme. The involvement of medical doctors – that is, activating the “professional authority” mechanism – provides a necessary condition for success.

## Discussion

There is nothing intrinsic about quality registers that makes them work in local clinical practice. Instead, it is important to understand what works for whom. Our realist evaluation drew attention to generative mechanisms, activities, and outcomes in specific contexts of heart failure care. We used a theory of professionalism in knowledge-based work [13] which makes it possible to understand the links between quality register initiatives and professionalism within the occupations; as part of organisational improvement and clinical practice. We argue that there is a significant tension between the different interests of these two logics, which makes the investments in quality registers more or less successful. Although the results in the present research are generated from the specific case of heart failure, we propose that the two logics are valid in other similar healthcare contexts having central roles of MDs. This discussion contrasts and compares our findings with other studies on the use of quality registers.

### Key roles in the programme

Previous research has generally argued that collaborative efforts between healthcare administrative management and professionals are difficult to organise [15]. Such relationships are often characterised by conflict and opposing interests. For instance, managers often make decisions that have an influence over the practice’s various activities, and at the same time medical doctors and nurses have a responsibility for patients’ treatment and care. This is a matter of risk, and the health professionals are responsible by law for their actions [16]. The same situation applies in the context of the present study. The logics of organisational improvement arising from political and administrative interests are not fully compatible with clinical practice logics. We have shown that it is very difficult for programmes based on organisational improvement logics to reach into the domain of the physician’s use of data. Mechanisms for using quality register data in this context arise from clinical practice, but external initiatives struggle for acceptance. An example is the troublesome double documentation the quality registers have led to. Recording patient information in medical records is a legal requirement’ and ensures quality and security in healthcare. Accordingly, implementation of quality registers meant a double documentation in the medical record as well as in the register. The mechanisms clearly indicate that a quality register is one thing, but daily documentation is something else. The problem is that the NQR programme did not bridge this chasm. This finding is corroborated by other studies of professional work, where work structures create barriers for bridging different competing logics [17].

One key issue that needs to be addressed is the involvement of medical doctors and their specific interests. This is relevant not only for how they process and use data from the SwedeHF register, but also for how they influence other contexts.

### Active involvement of medical doctors

A key focus in the NQR programme is to increase the use of data in clinical work. Much of this occurs in the context of using output data. The key role in the programme is the doctor who works in the clinical environment. The empirical results from SwedeHF show that improvements are made possible by the doctor’s group. Nurses working with registration are dependent on doctors who make assessments and target efforts towards patients [1].In the governance structure, policymakers and administrative staff claim that doctors play a decisive role in the development of the registers. Participants in improvement projects also refer to the goodwill of doctors to pursue initiatives that are in line with their interests. The apparent division between the improvement leaders and the medical doctors – although they had both used SwedeHF – meant that they did not talk about the register and its benefits in the same way. They used different expressions, which reinforced the gap between them. The logics of organisational improvement or clinical practice, thus, work in different directions. Similar findings have been found in previous research, where professionals seem to be stuck in their own way of reasoning and defend their professional autonomy [18]. An underlying explanation behind the “winning” logic is the health professional who is a strong leader, and has a central role in carrying out the work [19].

### Ambiguous roles and lack of decision support systems

What medical doctors do to develop “their own” context, using output data, is of particular interest. What we see here is a strong path dependence, as NQRs have historically been tightly linked to the medical profession’s competence [20]. This is also witnessed by the lack of mechanisms originating from the organisational improvement logic in the context of using output data. Thus, the integration of new ideas is very low. Rather, the use of data is a result of the professional’s identification with the register [8]. Previous research in RiksStroke (NQR for Stroke) with empirical data from a variety of settings (8 hospitals and 4 county councils) showed that the register itself did not initiate quality improvement in clinical practice [4]. Collaboration between local stakeholders such as persons in charge of the register, registers collecting the data and managers of the stroke units was important for quality improvement to occur.

### Medical doctors’ influence

In having a key role, the medical doctors not only influence the context which they occupy in everyday practice, but also other organisational contexts; that is, contexts where their involvement is more passive. Although others have higher levels of access to these contexts – registration is run by nurses, the governance structure is led by policy officials and administrators, and the improvement projects are organised by improvement experts – medical doctors’ influence is manifested through both passive and active ways of restricting activities. For instance, in the registration context there is the professional division in practice and, as such, this has prevented learning among professionals. Those who registered the SwedeHF data were most often nurses, and in some cases assistant nurses, due to a lack of resources in practice. The only time a doctor registered clinical data was in terms of a research project. There is a broad substantiated consensus among the respondents that SwedeHF is a medical register, which is why doctors are so central in making a difference when learning from the results. This finding is also highlighted in other studies of data registries and performance review systems [21, 22]. It further seems to be consistent across similar healthcare contexts [23]. Patterns of how the clinic treated its patients with heart failure became clearer when doctors carried out the registrations. Nurses could sometimes discover performance patterns, but because they had to transfer this knowledge to the doctors, the learning impact got lost along the way. Similar findings have been made in studies in RiksStroke [5]. The assistant nurses did not gain any knowledge insights at all, as they did not have the professional ability to identify these medical patterns. A competence hierarchy determines whether or not the learning opportunity is used, and this is maintained during the programme period. Similarly, within the governance structure, policy officials and administrators witness the difficulties of implementing new ideas as the medical profession opposes these initiatives.

### Future opportunities

What might be done to overcome the observed “flaws” in the registry system to improve the registry’s contributions to individual patient care, clinical program performance improvement and research? As has been argued in the paper, different logics can be characterised as “rules of the game” embedded in unconscious social norms that are part of work. One of the key issues is to look at the professionals who can bridge these different rules, so called hybrid professionals [24]. It is through them we may understand how to break the barriers. They are representatives of the medical profession, who relate not only to their clinician logic but also to the logic of organisational improvement. Thus, their everyday work is characterised by handling potentially conflicting different logics and mediate different interests. These hybrid professionals are supportive of bringing in organisational improvement logics to a clinician logic. Therefore, development of healthcare systems would benefit from finding ways how to identify and place hybrid professionals in leading positions. Another study supports the idea to emphasize the demands from local stakeholders and the specific end-users to increase to use of NQRs in clinical practice [25]. The study used empirical data from 9 different NQRs with a wide variety of indicators such as intervention indicators, diagnosis indicators, prevention indicators, palliative and psychiatry indicators. Generalisation of what works for whom in which context to all 100 registries must therefore be made with caution as the end-users differ a lot and so do their social norms.

### Limitations of the study and further research

Although we have validated our mechanisms through evaluations of causal explanation frameworks [26], other respondents might have highlighted other aspects. This would be an interesting continuation of this work, namely to conduct further studies with a higher proportion of clinicians who work with the NQR [27]. The external validity of the study is limited to contexts where there is high physician influence and traditional hierarchical structures, as in the sample from a university hospital. The results are also limited to registers concerning diagnosis and treatment specific indicators that are handled by a medical doctor. We saw that the programme rationale, with its focus on organisational improvement logic, had difficulties reaching into the very core functioning of this system. It would be interesting to validate these findings in smaller, non-university hospitals as well as in primary care.

Another significant question is if we can place the findings in an international context? Most healthcare systems in the western world are using data registers for monitoring, controlling and improving healthcare processes and performance [21]. The structure of these arrangements varies, in terms of characteristics of the patient group, healthcare processes and administrative set up for data registration. Therefore, generalizing must be done carefully. With its emphasis on the role of context and the ever-changing nature of how programmes are implemented and interacted with by their participants, realist evaluation is cautious about the possibilities of making general claims. With this in mind it is suggested that two logics of *organisational improvement* or *clinical practice* are more or less embedded in any register use. In addition, the mechanisms associated with each logic is proposed to enable effective use of registers, dependent on the context (i.e. *registration, use of output data, governance,* and *improvement projects)*. Our study is in this sense valid as a guiding support that enable others to understand what works for them in their own systems.

## Conclusions

What works for whom in what context when it comes to new forms of using register data in heart failure care? Our conclusion is that new ideas have difficulty reaching the core functioning of healthcare when based on a logic emanating from organisational improvement. Consequently, few or none of the mechanisms that should supposedly trigger new ways of using quality register data are able to be activated due to limited input from clinical practice.

## Supplementary information

**Additional file 1.** Description of data collection and sampling.

**Additional file 2.** Semi-structured interview guide 1 and 2.

**Additional file 3.** The research process.

## Data Availability

The datasets used and analysed during the current study are available from the corresponding author on reasonable request.

## Abbreviations

CP: Clinical practice
MD: Medical doctor
NQRs: Swedish national quality registers
OI: Organisational improvement
P4P: Pay for performance
PREM: Patient-reported experience measures
PROM: Patient-reported outcome measures
SALAR: The Swedish Association of Local Authorities and Regions
SwedeHF: Swedish Heart Failure Register

## Footnotes

1 In the presentation of our empirical data we use the term SwedeHF when this particular register is talked about and the abbreviation NQR when the respondents talk about national quality registers in general.

2 PROM – patient-reported outcome measures

3 PREM – patient-reported experience measures
