# Supplementary material for: The role of professional logics in quality register use: a realist evaluation
Source: BMC Health Serv Res. 2020 Feb 11;20:107. doi: 10.1186/s12913-020-4944-x (PMC7014753; doi:10.1186/s12913-020-4944-x)
Supplement: Supplementary file 1 — Additional file 1. Description of data collection and sampling. [file 12913_2020_4944_MOESM1_ESM.docx]

# Description of data collection and sampling

Eligible study participants were chosen through purposeful sampling, and the respective heads of department were provided with written information about the study and a request for permission to interview the clinical professionals. In the event of acceptance, individuals were contacted and informed about the study. The dates for the interviews were decided together with the individuals. All interviews were conducted at the respective workplaces, except for the interviews with representatives from the SwedeHF organisation, which were conducted at the researchers’ workplace. Informed consent was provided by all study participants prior to the interviews. Individual interviews lasted about 45–90 minutes. All interviews were recorded and transcribed verbatim. See Table A for a detailed overview of sampling.

Individuals were interviewed by means of a semi-structured interview guide (Guide 1) that consisted of five parts: (A) Business development, quality improvement strategies and implementation, and the role of SwedeHF; (B) clinical research and role of SwedeHF; (C) actors surrounding SwedeHF and their role; (D) patient-/user-participation in the work with SwedeHF; and (E) SwedeHF itself, its organisation and service. In the event of follow-up interviews, the guide consisted of the same parts but changes within each area were considered.

Furthermore, the two interviews with participants in and the organizer of the quality improvement project were conducted using a different semi-structured interview guide (Guide 2), which consisted of three parts: (A) Expectations of the quality improvement project (QIP) and the QIP’s actual setup; (B) experience of implementing the QIP; and (C) the effect of the QIP. Semi-structured questions for the third part were drawn from the clinical microsystem framework that is defined as the combination of people working together on a regular basis in processes with certain patterns for a specific purpose.

Table A. Overview of the sampling, N (interviews)=18.

| Organisational level | Scope | Role | Time point | | |
| --- | --- | --- | --- | --- | --- |
|  |  |  | 2013 | 2014 | 2015 |
| Macro | National, hospital | register coordinator, nurse | x |  |  |
|  | National, hospital | register manager, physician | x | x | x |
| Meso | county | head of healthcare service |  |  | x |
|  | *RCC¹ | leader for QI² project |  |  | x |
|  | hospital | medical information officer |  |  | x |
| Micro | hospital | head of unit, physician | x |  |  |
|  | hospital | head of department, physician | x |  |  |
|  | hospital | nurse, contact person for register | x |  | x |
|  | hospital | assistant nurse, registrar | x |  |  |
|  | hospital | physician, researcher, teacher,  physician responsible for SwedeHF | x |  | x |
|  | hospital | physician, researcher, teacher, member of steering group for SwedeHF | x |  | x |
|  | hospital | nurse, participant in quality assurance group for heart failure | x |  |  |
|  | *hospital | nurses’ contact persons for registers, participants in QI project |  |  | x |

¹RCC – Regional competence centre, ² QI – Quality improvement, * interviewed using Guide 2.
