# Supplementary material for: The role of professional logics in quality register use: a realist evaluation
Source: BMC Health Serv Res. 2020 Feb 11;20:107. doi: 10.1186/s12913-020-4944-x (PMC7014753; doi:10.1186/s12913-020-4944-x)
Supplement: Supplementary file 2 — Additional file 2. Semi-structured interview guide 1 and 2. [file 12913_2020_4944_MOESM2_ESM.docx]

# Semi-structured interview guide 1 and 2

(original in Swedish, translated by www.translate.google.com)

The development of some parts of the interview guide 1 were inspired by the *eHealth Value Framework for Clinical Adoption and Meaningful Use* from Lau et al.

(Lau F, Price M, BAssi J. Toward a Coordinated Electronic Health Record (EHR) Strategy for Canada. Draft White Paper, Version 3.1. In: School of Health Information Science UoV, editor. 2014. p. 1-22.)

How is the major investment in national quality registries utilized?

an interactive, learning evaluation

Thank you for taking the time to meet me / us for this research study.

The purpose of this research project is to carry out a number of evaluations to develop knowledge of the processes that lead to the effects that can be demonstrated by the investment in quality registers. The evaluations are expected to contribute with knowledge of barriers and conditions for utilization of registers as well as a better understanding of work and organizational forms, change processes and decisions that are important for a commitment of this scope to have maximum effect on the development of care and the ability to contribute to better and more equal health and quality of life in the population.

The approach is based on interactivity and learning between researchers, practitioners and patients and can take the form of analysis seminars. Therefore, we ask you to be able to contact you again at a later date.

The interview will cover 5 general themes, which are as follows:

A) Business development / quality improvement: strategies and implementation

B) Clinical research and NKR's role in this

C) Actors around NKR

D) Patient / user participation in work with NKR

E) NKR operations: design / service of NKR

The interviews will be recorded and transcribed and analysed by our research group at Jönköping Academy, Jönköping School of Health. Your answers will not be shared with anyone outside the research group without being anonymized. Participation is voluntary and you can cancel at any time without explanation.

PAUSE

- for questions and comments

• Before we move on, do you have any questions you would like to ask?

• Ask if the person wants to continue with the interview

• Check that the consent form has been read - understood - signed?

Recording begins:

GUIDE 1 semi-structured questions:

Welcome name, title, function, hospital, etc.

An introductory question:

Would you like to tell me about your connection to NKR, then when you work and how you work with NKR and how it has changed over time?

A) Business development / improvement work: strategies and implementation and its effect

1. Tell us how KR can actually support business development / quality improvement - example? What opportunities do you see?

2. Tell us how you work in practice using KR in business development / quality improvement

3. Problems - tell us about problems you see - example?

- Can you describe how decision-making of the hospital's goals and tasks is accomplished, are there explicitly such applicable quality of care and improvement work?
- How would you describe the policy regarding quality work at your hospital?
- Can you describe the process of how managers evaluate and adjust care processes?
- Can you describe how the business reports its results to the public?
- Atmosphere in hospital / unit: Family CEO task oriented
- Organizational structure - horizontal vs. hierarchical; centralized vs. decentralized
- What role a manager in business development – characteristics

B) Clinical research

1. What role does clinical research have here, how do you see this and how are KR examples used?

2. How do you view the relationship between clinical research and improvement work?

3. Opportunities - tell us about opportunities you see - example?

4. Problems - tell us about problems you see - example?

- Are there designated resources for quality improvement?
- How would you describe the management team
- How would you describe the company's policy to provide its staff with the conditions to promote quality and participate in improvement work? -> further education in quality improvement (who at what level)
- How large is the use of register data among the staff, who uses it most?

*Improvement knowledge = Professional knowledge (subject knowledge, personal skills, values, ethics) + improvement knowledge (systems, variation, psychology of change, learning-driven change work)*

*-Improvement of diagnosis, nursing, treatment and rehabilitation + improvement of working methods, processes and systems === increased value for those care / caregivers are available for*

C) Actors around NKR: their activities and their effect / consequences thereof

1. Tell the players around NKR! Which are important?

2. Why?

3. Opportunities - tell us about opportunities you see - example?

4. Problems - tell us about problems you see - example?

- Micro: Register holders and clinical professionals, direct users (input data), register service
- Meso: The organization, business developers, etc, Implementation process
- Macro: standards (NI, NF), laws, politics, funding, patient associations
- How do these activities affect your work with NKR?
- How do you collaborate with these actors?
- How do you collaborate with hospital management, business improvers, business economists, etc?
- How do you collaborate with other county councils?
- How do you collaborate with SKL, the National Board of Health and Welfare?
- Are there other actors that have not been mentioned so far but that are important?

D) Patient / user participation in work with NKR and its effect

1. Can you tell us how you look at patient participation in work with NKR?

2. Opportunities - tell us about opportunities you see - example?

3. Problems - tell us about problems you see - example?

- Do you know how the population / patients look at quality records?
- Can you describe how patients can be involved in quality work?
- Patients are involved in ...
- ... The development of quality criteria / standards / protocols
- ... Design / organization of processes
- ... Quality Committees
- ... Quality improvement projects
- ... Discussion of the results of the projects for quality improvement

E) NKR operations: design / service of NKR and its effect

1. Can you tell us how you look at NKR's design and its service?

2. Opportunities - tell us about opportunities you see - example?

3. Problems - tell us about problems you see - example?

- How do you see the usability of NKR? Is it easy and time efficient to use?
- Are measures and indicators relevant to follow up?
- Is data quality high?
- How does data entry / double documentation work?
- How does it work to extract data / feedback etc? Real-time feedback
- How does security work?
- Do you do user surveys?
- Do you get immediate help with technical problems?
- Do you get user training?
- What changes / improvements would you need to use NKR more?

And some closing questions:

How do you want us to continue our work to feedback the results of the investigation and to delve deeper into interesting questions that will appear in the ongoing investigative work?

Now we have talked about a lot. What do you think is crucial for the work with NKR to be successful and well-integrated and used in the future?

Is there anything you would like to add yourself that I haven't asked?

Thank you for participation!

GUIDE 2 semi-structured questions:

1. Introductory & transition questions.

- Why do you think the education has been developed?
- How did you get in touch with the education?
- Who decided you just wanted to join?

For participants of Quality Improvement Project, questions 2-3 only

1. If we think about the time before the training, when it was decided that you would attend:

- Do you remember what you thought the training would provide? What expectations did you have?
- Did you talk about these expectations?
- In what contexts did you talk about it?
- What did your boss do?

1. Research question - NKR education:

- How does education go about in practical terms? How is it organized?
- What conditions do you need to be able to participate in such a training?
- What are the requirements for you to participate in the education?
- Are you used to such an arrangement?

For organizer of Quality Improvement Project, questions 4-5 only

4. The improvement project and expectations

- Can you tell us about the improvement project - how is it planned and what is included? (information, content, purpose)
- What past experiences do you have of such improvement projects?
- Did you consider other arrangements? Why? (theories, frameworks)
- How did you do to promote learning in the improvement project?
- When you started the improvement project - what expectations did you have?
- What was your interest in the improvement project? (National / director / Cast).

5. Experiences of implementing the improvement project

- How do you think the arrangement worked? What worked well / less well? Why? (relevance, time, quality, content etc.)
- Did you have the necessary prerequisites to carry out your improvement project? How was the support from different directions? (participating organizations, within your own organization, nationally) (obstacles and opportunities) (leadership and organizational support)
- What support and conditions would you like if you arranged a similar project?
- How can your improvement project be improved?

1. Research question - Experience:

- We are curious about what the education has led to.
- What did you learn during the training? Tell!

1. Operationalisations:

- Has it affected how you lead & collaborate in the work in any way? How? (control / moral action) + (P as in People)
- Has your view of your business changed in any way? How? (Particular / Holistic)
- Has it affected your view of why you should work with the register? (P as in Purpose + P as in Problem)
- Has it affected the view of the patients (P as in Patient groups)
- Has the education / knowledge affected how you collaborate? The team / professions / management / patients (Power / Equality)

1. Research question - Effects:

If we now look at the results as they are compiled in NKR;

- Are you better today? (operationalization: P as in Patterns)
- Have there been any changes in the business? Who then and how big are they?
- How do you explain it?
- What does this mean for your patients? (Operationalization: P as in Patient Groups)
- What could be better?

1. Ending questions:

- How do you use the register today if you compare with the time before the training?
- What is the biggest and most important change?
- Does education meet your expectations?
- What do you think about the future development?

I think I have answered many of my questions and want to end by asking if there is something you want to add or clarify before we finish the interview?

Thank you for your participation!
