# Supplementary material for: The role of professional logics in quality register use: a realist evaluation
Source: BMC Health Serv Res. 2020 Feb 11;20:107. doi: 10.1186/s12913-020-4944-x (PMC7014753; doi:10.1186/s12913-020-4944-x)
Supplement: Supplementary file 3 — Additional file 3. The research process. [file 12913_2020_4944_MOESM3_ESM.docx]

# The research process

A detailed description of how theory and methodology inform each part of the research process.

- Preliminary literature review
- Decision on data collection for the NQR programme as a whole

Data collection 2013–2015

- Case selections in research group
- Research approach and sample of this SwedeHF case study

Selection of data for this SwedeHF case study

**Third round of data analysis**

- Categorising mechanisms by two logics of professionalism: organisational improvement or clinical practice including six aspects
- Explaining mechanisms in relation to their contexts and outcomes

**Final round of data analysis**

- Verifying consistency of findings

- Checking for possible alternative explanations from literature

- Discussion in research group

Generation of propositions and practical implications

- Realist evaluation as an overarching methodology to identify context-mechanism outcome configurations [9]
- Theory of professionalism to explain why the initiatives of the NQR program regarding SwedeHF succeed or fail in clinical practice [13]

Categorisation informed by Sztompka [12].

Methodological literature review

**Second round of data analysis**

Identifying contexts, mechanisms, activities and outcomes in the empirical data

Design of analytical framework and process

**First round of data analysis**

Thematic categorisation of contextual factors that facilitate or govern the use of SwedeHF in clinical practice
